# The Complete Mitochondrial Genomes of *Penthe kochi* (Coleoptera: Tetratomidae) with Its Phylogenetic Implications

**DOI:** 10.3390/cimb46100641

**Published:** 2024-09-26

**Authors:** Bowen Ouyang, Yingying Li, Jieqiong Wang, Zhonghua Wei, Aimin Shi

**Affiliations:** College of Life Sciences, China West Normal University, Nanchong 637009, China

**Keywords:** Tenebrionoidea, Tetratomidae, *Penthe*, mitogenome, phylogeny

## Abstract

To explore the mitogenome characteristics of Tetratomidae and the phylogenetic position of this family in Tenebrionoidea, the mitogenome of *Penthe kochi* Mařan, 1940 was sequenced, annotated, and analyzed. The *P. kochi* mitogenome is consistent with Tenebrionoidea species in gene length, genomic organization, codon usage, and secondary structures of transfer genes (tRNAs). Most protein-coding genes (PCGs) originate with a typical ATN start codon, except *nad1* and *nad3,* which start with TTG. In total, 10 PCGs are terminated with complete stop codon TAA and TAG, while *cox1*, *cox2*, and *nad 4* contain an incomplete stop codon T-. Among the 13 PCGs, *nad2* (Pi = 0.282) has the most diverse nucleotide composition, and *cox2* is the most conserved gene with the lowest value (Pi = 0.154). The Ka/Ks ratio of *cox1* (0.076) and *cox2* (0.124) has a lower value. All the tRNAs can be folded in a typical clover-leaf secondary structure, except *trnS1*, which lacked a dihydrouridine arm. And phylogenetic analyses were performed based on 13 PCGs using the Bayesian inference (BI) method. The results showed that the clade of Tenebrionoidea was well separated from the outgroups, and Tetratomidae and Mycetophagidae were not well resolved. Phylogenetic analyses with more mitogenome samplings are needed to resolve the phylogeny of Tenebrionoidea.

## 1. Introduction

The family Tetratomidae, including 13 genera over 150 species in five subfamilies [1], is a small group in the superfamily Tenebrionoidea. The tetratomid species are widely distributed in the Palearctic, Oriental, Sino-Japanese, Australian, Oceanian, Afrotropical, and Nearctic regions. To date, eight genera and twenty-two species in five subfamilies have been recorded from China, primarily distributed in the southwest and southeast [2,3,4,5,6,7,8,9,10]. Adults are frequently found on the surface of Polyporaceae or Tricholomataceae at night, and their larvae feed internally on hyphal tissue [1].

Although many taxonomists have made important contributions to the Tenebrionoidea classification during phylogenetic analyses based on morphological characteristics and molecular data, the problems of the overall classification remain unsettled. Based on the morphological characteristics, Tenebrionoidea (26 families) was divided into five lineages, one of which was composed of Tetratomidae, Melandryidae, Mordellidae, and Ripiphoridae [11]. Based on the molecular data, Hunt et al. performed a comprehensive phylogenetic analysis of beetles inferred from three genes (*16S*, *18S*, *cox1*); the results suggested that the 23 sampled families of Tenebrionoidea formed a clade [12]. Mckenna and Farrell constructed a Timetree of life of Coleoptera based on morphological characteristics, fossils, and molecular data, and the results suggested that the 21 sampled families of Tenebrionoidea formed a clade with the origin of Tenebrionoidea being ca. 236 Ma [13]. Gunter et al. reconstructed the phylogenetic tree of 24 tenebrionoid families based on four genes (*ssu*, *lsu*, *rrnL*, *cox1*), and the results showed that topology included four lineages [14]. Then, McKenna et al. reconstructed the phylogenetic tree of Coleoptera based on eight nuclear genes; the results exhibited that Tenebrionoidea included the 28 sampled families and Lymexyloidea was recovered within Tenebrionoidea [15]. And then, McKenna et al. inferred the phylogeny of beetles using 4818 genes for 146 species [16]; the results suggested that the phylogenetic position of Lymexylidae in Tenebrionoidea is suitable. However, Cai et al. used 68 single-copy nuclear protein-coding genes of 129 extant families to explore beetle evolution, and the results suggested that Lymexyloidea is a sister group of Tenebrionoidea [17]. However, Tetratomidae is not included in these studies. In Tenebrionoidea, the phylogenetic position of Tetratomidae is rather unclear.

Recently, the mitogenome emerged as a valuable source for higher-level phylogenetic analysis [18,19,20,21]. In this study, the first complete mitochondrial genome for Tetratomidae, the mitogenome of *Penthe kochi* Mařan, 1940, was sequenced, annotated, and comparatively analyzed. Moreover, phylogenetic analyses based on the Bayesian inference (BI) method was carried out to assess the phylogenetic position of Tetratomidae in Tenebrionoidea and to contribute to further understanding the phylogenetic relationships of each group of Tenebrionoidea.

## 2. Materials and Methods

### 2.1. Sampling, Identification, and DNA Extraction

The specimens of *Penthe kochi* Mařan, 1940 were collected from Dayaoshan Mountains, Jinxiu County, Guangxi Zhuang Autonomous Region, China, on 11 February 2021. Specimens were immediately preserved in 95% ethanol in the field after they were collected and then stored at −24 °C in laboratory. The specimens were examined using Olympus SZX10 and were identified based on the morphological characteristics described by Mařan [22]. Genomic DNA was extracted from the legs and thoracic muscle tissue. Next-generation sequencing and the assembly of mitogenome were performed by Beijing Aoweisen Gene Technology Co., Ltd. (Beijing, China).

### 2.2. Mitogenome Assembly, Annotation and Analysis

A whole genome shotgun strategy was used based on the Illumina HiSeq platform when the total genome DNA was quantified. Then, sequencing was performed with a strategy of 150 bp paired-end reads. The assembler MITO-bim was used for mitogenome assembly [23]. To check the correctness of the assembly, clean data were manually mapped on the mitochondrial scaffold using Geneious 11.0.2 software [24]. The 13 protein-coding genes (PCGs) and two ribosomal RNA genes (rRNAs) were identified using Geneious 11.0.2. The 22 transfer RNA genes (tRNAs) were re-identified using a tRNAScan-SE server v 1.21 and MITOS WebSever [25,26]. The mitogenome map was illustrated using the online tool Organellar Genome DRAW (OGDRAW; https://chlorobox.mpimp-golm.mpg.de/OGDraw.html, 11 September 2024) [27]. In an A + T-rich region, the tandem repeat elements were identified using an online tool, Tandem Repeats Finder [28]. The relative synonymous codon usage (RSCU) was calculated by Mega X [29]. The nucleotide diversity (Pi) and the ratio of nonsynonymous/synonymous (Ka/Ks) PCGS were calculated with DnaSP v 5 [30].

### 2.3. Phylogenetic Analysis

The mitochondrial genome of 33 species from 15 families of Tenebrionoidea were selected as ingroups (Table 1), and mitochondrial genomes of Lymexylidae species were chosen as the outgroups, as they are phylogenetically distant from Tenebrionoidea in the Coleoptera [17,31]. The nucleotide sequences (13 PCGs) of 33 mitogenomes were aligned using ClustalW and trimmed using trimAl v 1.2 [32,33]. The best-fit model was determined by ModelFinder based on Bayesian information criterion. The phylogenetic trees were constructed using PhyloSuite v 1.2.2 [34], based on the Bayesian inference (BI) method. Bayesian analyses were run with two independent chains spanning 2,000,000 generations, four Markov chains, with sampling at every 100 generations and a burn-in period of 0.25 for each chain. The phylogenetic trees were edited and visualized by Figtree v 1.4.3.

## 3. Results and Discussion

### 3.1. Genome Organization and Base Composition

**Figure 1:**
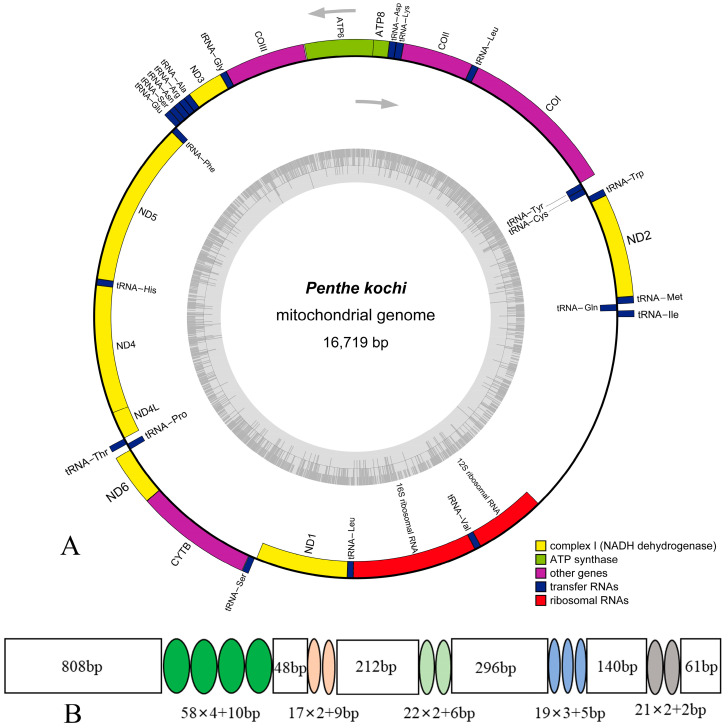
(**A**) The mitogenome map of *Penthe kochi*; (**B**) the organization of the A+T-rich region in the *P. kochi* mitogenome.

The mitogenome of *P. kochi* is deposited in GenBank with accession number (ON113044). The mitogenome sequence is 16,719 bp in length and encodes 37 typical mitochondrial genes: 13 protein-coding genes (13 PCGs), two ribosomal RNA genes (2 rRNAs), 22 transfer RNA genes (22 tRNAs), and an A + T-rich region (control region, CR) (Figure 1). The gene arrangement of the *P. kochi* mitogenome is identical to the hypothetical ancestral insect pattern [43]: 14 genes (4 PCGs, 8 tRNAs, and 2 RNAs) are N-strand, and the other genes (9 PCGs and 14 tRNAs) are J-strand (Table 2). This mitogenome contains 11 overlapping genes, and the longest overlap is 7 bp between *nad4* and *nad4l*. In tRNAs, the gene overlap probably represents the lower evolutionary constraints [39,44]. In the *P. kochi* mitogenome, intergenic nucleotides range from 1 to 34 bp, with the longest region located between *trnW* and *trnC*.

The base composition of the mitogenome of *P. kochi* is A (40.97%), C (11.69%), G (7.70%), and T (39.63%) (Table 2 and Table S2). The nucleotide composition has a high A/T bias (80.60%), the same as other beetles [42,45,46,47,48], and exhibits a positive AT-skew (0.02) and a negative GC-skew (−0.21), indicating that A and C are more abundant than T and G.

### 3.2. Protein-Coding Genes and Codon Usage

The total length of 13 protein-coding genes (PCGs) is 11,130 bp, approximately accounting for 66.57% of the entire mitochondrial genome. In total, 4 of the 13 PCGs (*nad1*, *nad4*, *and4L*, and *nad5*) are located on the minority strand (N-strand), and the other nine (*nad2*, *cox1*, *cox2*, *atp8*, *atp6*, *cox3*, *nad3*, *nad6*, and *cytb*) are located on the majority strand (J-strand) (Table 2). The A/T nucleotide composition of PCGs is 78.99%, exhibiting a high A/T bias (Table S1). The AT-skew of the entire PCGs is −0.14. Among the 13 PCGs, *nad5* (1713 bp) and *atp8* (156 bp) were found to be the largest and smallest genes, respectively. Most PCGs originated with a typical ATN (ATA, ATT, ATG) start codon, except *nad1* and *and3,* which started with TTG. Moreover, with the exception of *cox1*, *cox2,* and *nad 4* that contain an incomplete stop codon T-, the other 10 PCGs were terminated with complete stop codon TAA (*nad6*, *cytb*, *nad4l*, *nad5*, *atp8*, *atp6*, *cox3*, and *nad2*) and TAG (*nad1*, *nad3*). In insects, lots of mitochondrial genes have incomplete stop codons, which is consistent with previous research [49].

**Figure 2:**
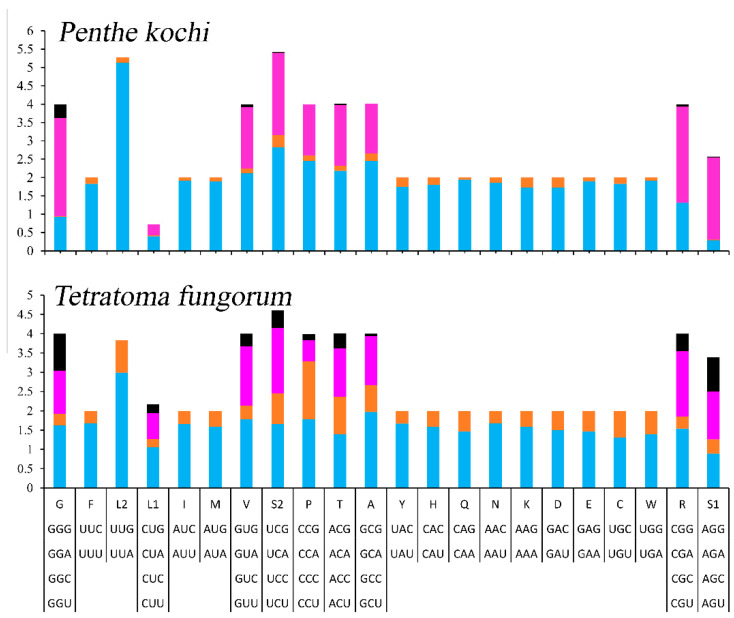
The relative synonymous codon usage of mitogenomes of Tetratomidae.

The relative synonymous codon usage (RSCU) of the mitogenomes was calculated and is exhibited (Figure 2). The 13 PCGs contain 3699 codons, excluding stop codons (Table S2), and the most frequently used codons are UUA (508), AUU (429), UUU (311), and AAU (214). Accordingly, L2, I, F, and N are the most frequently used amino acids, accounting for 13.73%, 11.60%, 8.41%, and 5.78% of the total amino acids, respectively.

To determine the differences between Penthinae (*P. kochi*) and Tetratominae (*T. fungorum*), the authors compared the length, acid composition, and RSCU of PCGs. The whole mitogenome of *P. kochi* (16,719) is longer than the *T. fungorum* (15,089). In the 13 PCGs, the size of *nad2*, *cox2*, *nad5*, *nad4l*, *nad6*, *cytb,* and *nad1* is longer in *P. kochi* than in *T. fungorum*, but *cox1* is the opposite (Table S3). And the length of the *rrnL* and *rrnS* of *P. kochi* (1285 bp and 735 bp) is shorter than that of *T. fungorum* (1364 bp and 768 bp). In the *P. kochi* mitogenome, the CUG codon used for L1, the CCG codon used for P, the CGC codon used for R, and the GCG codon used for A are absent (Figure 2); however, their stability requires further investigation. In Penthinae (*P. kochi*) and Tetratominae (*T. fungorum*), the amino acid composition and proportion are similar.

**Figure 3:**
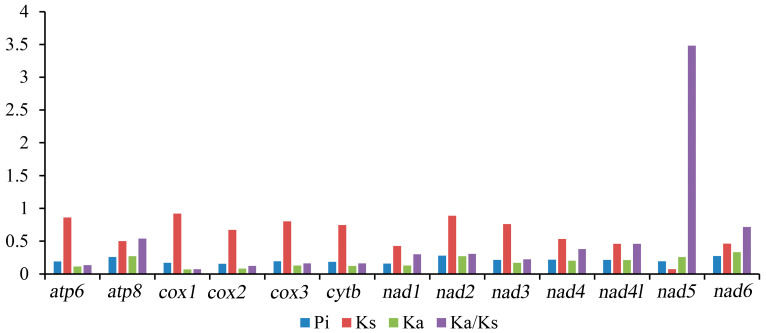
The Pi, Ka, Ks, and Ka/Ks of the 13 PCGs of mitogenomes in Tetratomidae. Pi. Nucleotide diversity, Ka. Nonsynoymous, Ks. Synoymous, Ka/Ks. Nonsynoymous/Synoymous.

In Tetratomidae, the nucleotide diversity (Pi) of 13 PCGs ranged from 0.154 (*cox2*) to 0.282 (*nad2*) (Figure 3, Table S1). Among the 13 PCGs, the gene *nad2* (Pi = 0.282) was the most diverse nucleotide, followed by *nad6* (Pi = 0.277) and *atp8* (Pi = 0.260). In contrast, the gene *cox2* was the most conserved gene with the lowest value (Pi = 0.154).

The ratio of nonsynonymous/synonymous (Ka/Ks) of PCGs was also calculated, which is representative of the evolutionary rate [50]. The Ka/Ks ratio of *nad5* (3.484) is distinctly larger than one, indicating that this gene is under positive selection [51], while the other 12 PCGs are under purifying selection (Figure 3; Table S4). Among them, *cox1* (0.076) and *cox2* (0.124) have a lower value. These data suggest that the genes *cox1* and *cox2* can be used as barcodes for deducing the phylogenetic relationships of Tetratomidae.

### 3.3. Transfer and Ribosomal RNA Genes

**Figure 4:**
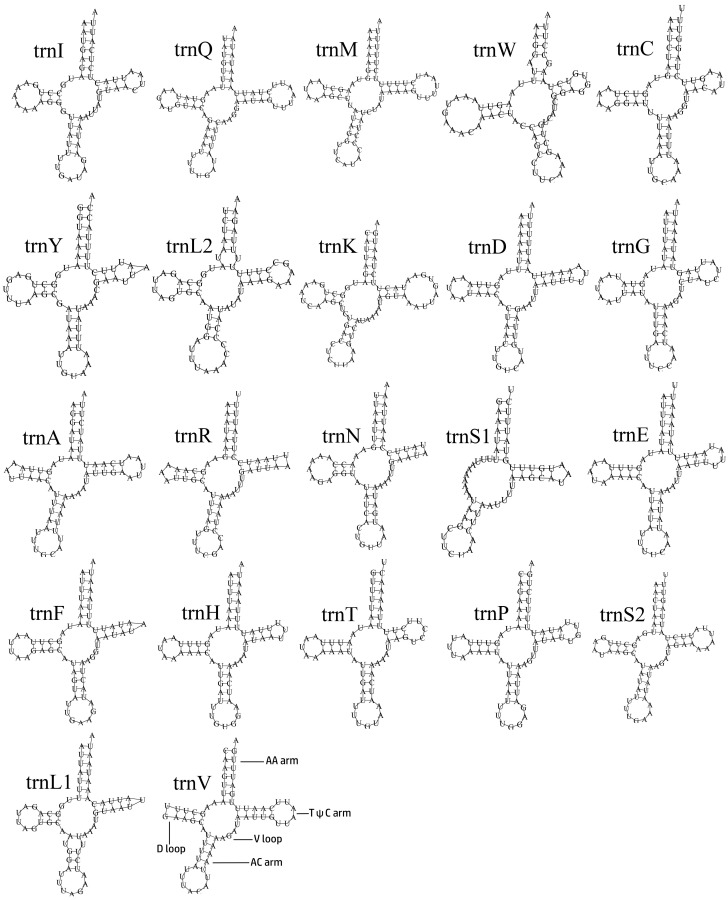
The predicted secondary structures of tRNAs in the mitogenomes of *Penthe kochi*.

The transfer RNA genes (tRNAs) are 1416 bp in total length, with a high A/T bias (A + T content is 80.08%). The AT-skew of whole tRNAs is 0.03 (Table S1). The tRNAs range from 58 to 71 bp in length. All the tRNAs can be folded in the typical clover-leaf secondary structure, except trnS1, which lacked a dihydrouridine arm (Figure 4), which is a common phenomenon for insects [52,53,54,55,56]. In the whole mitogenome, the longest intergenic nucleotide (34 bp) is located between *trnC* and *trnW*.

The lengths of *rrnL* and *rrnS* are 1285 bp and 735 bp, respectively. The A + T content is 82.52%, with a negative AT-skew (−0.04) and positive GC-skew (0.33). The *rrnL* subunit is located between *trnV* and *trnL1*, and the *rrnS* is located at the *trnV* and A + T-rich region.

### 3.4. A + T-Rich Region

It is well known that the A + T-rich region (control region, CR) acts in transcription initiation, transcription elongation, and DNA replication [57,58]. This non-coding region is 2119 bp in total length, located between *rrnS* and *trnI*. The A + T content is 87.54% (Table S5), with positive AT-Skew (0.04) and negative GC-skew (−0.39). There are five tandem repeat sequence units in this region, of which the length and position are provided (Figure 1).

## 4. Phylogenetic Analysis

**Figure 5:**
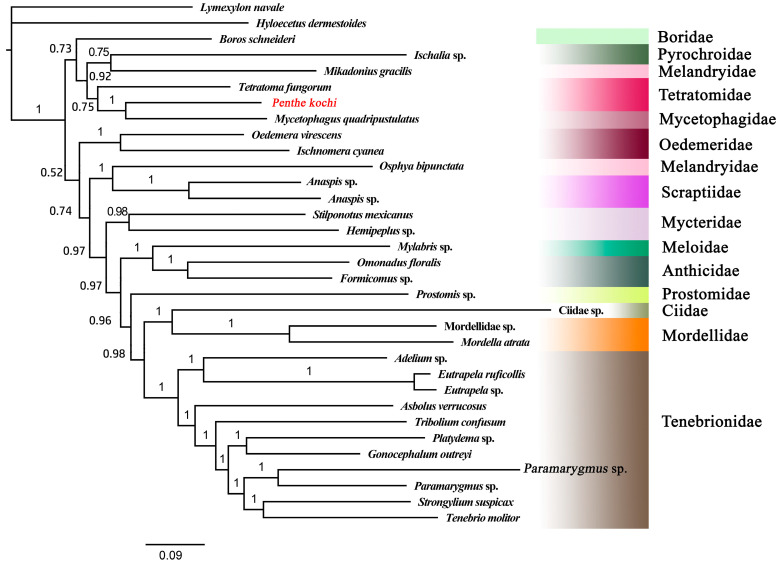
The BI tree based on 13 PCGs of Tenebrionoidea. The value at each branch shows posterior probability. The target species is displayed in red font.

The BI tree was constructed based on the best-fit model GTR + F+ I + G4. Based on the BI tree topology, the monophyly of Tenebrionoidea was again confirmed, as all the tenebrionoid species converged together as an independent clade with high-value support (PP = 1) (Figure 5). The Tetratomidae, Boridae, Mycetophagidae, Melandryidae, and Pyrochroidae converged together as an independent clade with high support (PP = 0.98), which formed a sister group of other tenebrionid families. All the Tenebrionidae species formed a clade. The Meloidae was close to Anthicidae and formed a clade. And Ciidae and Mordellidae were clustered together and formed a sister group of Tenebrionidae with high support (PP = 1). The results also showed that the Melandryidae is not monophyletic. So, the interrelationships among the families of Tenebrionoidea still require more data to be determined completely. These questions will be well addressed in the future when sufficient numbers of complete mitogenomes of Tenebrionoidea species are accumulated.

## 5. Conclusions

In the present study, the mitochondrial genome of *Penthe kochi*, which is the first complete mitogenome of Penthinae, was sequenced, annotated, and analyzed. It was found to be consistent with the known mitogenome of Tenebrionoidea in genomic organization, the codon usage of 13 PCGs, A + T biased base composition, and the secondary structures of tRNAs. Among the 13 PCGs, the gene *nad2* (Pi = 0.282) is the most diverse nucleotide, and *cox2* (Pi = 0.154) is the most conserved gene. The A + T-rich region has five tandem repeat sequence units with different lengths. Phylogenetic analyses based on 13 PCGs from 33 species show that Tetratomidae is close to Mycetophagidae.

## Figures and Tables

**Table 1:** The mitogenomes of Tenebrionoidea and outgroups were used for phylogenetic analyses.

Family	Sufamily	Species	GenBank No.	References
Tetratomidae	Penthinae	*Penthe kochi*	ON113044	This study
Tetratomidae	Tetratominae	*Tetratoma fungorum*	NC_036276	[35]
Mordellidae	Mordellinae	*Mordella atrata*	NC_013254	[36]
Mordellidae		Mordellidae sp.	JX412844	[31]
Mycteridae	Hemipeplinae	*Hemipeplus* sp.	JX412852	[31]
Mycteridae	Lacconotinae	*Stilponotus mexicanus*	JX412811	[31]
Mycetophagidae	Mycetophaginae	*Mycetophagus quadripustulatus*	HQ232824	[37]
Anthicidae	Anthicinae	*Formicomus* sp.	JX412857	[31]
Anthicidae	Anthicinae	*Omonadus floralis*	HQ232825	[37]
Boridae	Borinae	*Boros schneideri*	HQ232823	[37]
Meloidae	Meloinae	*Mylabris* sp.	JX412732	[31]
Oedemeridae	Oedemerinae	*Oedemera virescens*	HQ232826	[37]
Oedemeridae	Oedemerinae	*Ischnomera cyanea*	JX412790	[31]
Prostomidae	Prostominae	*Prostomis* sp.	JX412787	[31]
Pyrochroidae	Ischaliinae	*Ischalia* sp.	HQ232827	[37]
Scraptiidae	Anaspidinae	*Anaspis* sp.	HQ232806	[37]
Scraptiidae	Anaspidinae	*Anaspis* sp.	JX412856	[31]
Melandryidae	Melandryinae	*Mikadonius gracilis*	JX412823	[31]
Melandryidae	Melandryinae	*Osphya bipunctata*	JX313675	[31]
Ciidae		Ciidae sp.	JX412846	[31]
Tenebrionidae	Pimeliinae	*Asbolus verrucosus*	NC_027256	[38]
Tenebrionidae	Tenebrioninae	*Eutrapela* sp.	JX412754	Unpublished
Tenebrionidae	Lagriinae	*Adelium* sp.	FJ613422	[39]
Tenebrionidae	Lagriinae	*Eutrapela ruficollis*	HQ232805	[37]
Tenebrionidae	Tenebrioninae	*Tenebrio molitor*	NC_024633	[40]
Tenebrionidae	Tenebrioninae	*Tribolium confusum*	NC_026702	[41]
Tenebrionidae	Blaptinae	*Gonocephalum outreyi*	KU236386	[42]
Tenebrionidae	Alleculinae	*Paramarygmus* sp.	JX412775	[31]
Tenebrionidae	Diaperinae	*Platydema* sp.	JX412842	[31]
Tenebrionidae	Stenochiinae	*Strongylium suspicax*	JX412780	[31]
Tenebrionidae		*Paramarygmus* sp.	JX412808	[31]
Lymexylidae (outgroups)	Hylecoetinae	*Lymexylon navale*	KX087311	Unpublished
	Melittommatinae	*Hyloecetus dermestoides*	HQ232820	[37]

**Table 2:** Summary of the characteristics of the mitogenome of *Penthe kochi*.

Genes	Strand	Position		Size	Start Condon	Stop Codon	IGN	Anticodon
		from	to					
*trnI*	J	1	65	65			−	GAT
*trnQ*	N	63	131	69			0	AAG
*trnM*	J	132	200	69			0	CAT
*nad2*	J	201	1208	1008	ATA	TAA	−2	
*trnW*	J	1207	1273	67			34	ACA
*trnC*	N	1308	1368	61			0	GCA
*trnY*	N	1369	1431	63			<2	GAA
*cox1*	J	<1433	2966	>1534	?/?/?	T(AA)	0	
*trnL2*	J	2967	3030	64			0	AAA
*cox2*	J	3031	3718	688	ATT	T(AA)	0	
*trnK*	J	3719	3789	71			−1	CAA
*trnD*	J	3789	3853	65			0	GAC
*atp8*	J	3854	4009	156	ATT	TAA	−7	
*atp6*	J	4003	4674	672	ATG	TAA	5	
*cox3*	J	4680	5468	789	ATG	TAA	−1	
*trnG*	J	5468	5530	63			0	ACC
*nad3*	J	5531	5884	354	TTG	TAG	−2	
*trnA*	J	5883	5947	65			−1	AGC
*trnR*	J	5947	6009	63			−1	ACG
*trnN*	J	6009	6071	63			0	GAA
*trnS1*	J	6072	6129	58			0	ACA
*trnE*	J	6130	6193	64			−1	AAC
*trnF*	N	6193	6256	64			0	GAA
*nad5*	N	6257	7969	1713	ATT	TAA	0	
*trnH*	N	7970	8032	63			0	GAG
*nad4*	N	8033	9365	1333	ATG	T(AA)	−7	
*nad4l*	N	9359	9646	288	ATG	TAA	2	
*trnT*	J	9649	9711	63			0	AGA
*trnP*	N	9712	9775	9775			2	AGG
*nad6*	J	9778	10,284	507	ATT	TAA	−1	
*cytb*	J	10,284	11,420	1137	ATG	TAA	1	
*trnS2*	J	11,422	11,486	65			17	AGA
*nad1*	N	11,503	12,453	951	TTG	TAG	0	
*trnL1*	N	12,454	12,515	62			0	AAG
*rrnL*	N	12,516	13,800	1285			0	
*trnV*	N	13,801	13,865	65			0	AAC
*rrnS*	N	13,866	14,600	735			0	
A + T-rich region		14,601	16,719	2119			0	

IGN = intergenic nucleotide; J = the majority strand; N = the minority strand; and ? = not determined.

## Data Availability

The complete mitogenome is available at NCBI (ON113044).

## Supplementary Materials

The following supporting information can be downloaded at: https://www.mdpi.com/article/10.3390/cimb46100641/s1, Table S1. Nucleotide characteristics of the mitogenome of *Penthe kochi*; Table S2. The codon count and RSCU of the mitogenome of *Penthe kochi;* Table S3. The length of 13 PCGs in Tetratomidae species; Table S4. mitogenomeThe nonsynonymous (Ka) and synonymous (Ks) of *Penthe kochi* mitogenome; Table S5. The A + T content of the Tetratomidae; Figure S1. The nucleotide diversity of 13 PCGs of *Penthe kochi.*
