# Supplementary material for: The Complete Mitochondrial Genomes of Penthe kochi (Coleoptera: Tetratomidae) with Its Phylogenetic Implications
Source: Curr Issues Mol Biol. 2024 Sep 26;46(10):10795–805. doi: 10.3390/cimb46100641 (PMC11506331; doi:10.3390/cimb46100641)
Supplement: Supplementary file 1 [file cimb-46-00641-s001.zip › cimb-3188741-supplementary/Supplementary Materials_1.pdf]

Table S1. Nucleotide characteristics of the mitogenome of *Penthe kochi*.

	Size (bp)	A	T	G	C	A + T	G + C	AT-skew	GC-skew
Full genome	16,719	40.97%	39.63%	7.70%	11.69%	80.60%	19.40%	0.02	-0.21
PCGs	11,130	34.14%	44.85%	10.46%	10.55%	78.99%	21.01%	-0.14	-0.0043
rRNAs	2020	39.50%	43.02%	11.63%	5.84%	82.52%	17.48%	-0.04	0.33
tRNAs	1416	41.24%	38.84%	11.44%	8.47%	80.08%	19.92%	0.03	0.15
CR	2119	45.73%	41.81%	3.78%	8.68%	87.54%	12.46%	0.04	-0.39

CR = A + T rich region.

Table S2. The codon count and RSCU of the mitogenome of *Penthe kochi*.

Codon	Count	RSCU	Codon	Count	RSCU	Codon	Count	RSCU
UUU(F)	311	1.83	CCC(P)	5	0.16	AAA(K)	95	1.73
UUC(F)	28	0.17	CCA(P)	43	1.39	AAG(K)	15	0.27
UUA(L2)	508	5.13	CCG(P)	0	0	GAU(D)	55	1.72
UUG(L2)	14	0.14	ACU(T)	86	2.18	GAC(D)	9	0.28
CUU(L1)	40	0.4	ACC(T)	6	0.15	GAA(E)	74	1.9
CUC(L1)	3	0.03	ACA(T)	65	1.65	GAG(E)	4	0.1
CUA(L1)	29	0.29	ACG(T)	1	0.03	UGU(C)	32	1.83
CUG(L1)	0	0	GCU(A)	74	2.45	UGC(C)	3	0.17
AUU(I)	429	1.91	GCC(A)	6	0.2	UAG(W)	86	1.91
AUC(I)	21	0.09	GCA(A)	41	1.36	UGG(W)	4	0.09
AUA(M)	253	1.89	GCG(A)	0	0	CGU(R)	18	1.31
AUG(M)	15	0.11	UAU(Y)	139	1.74	CGC(R)	0	0
GUU(V)	77	2.11	UAC(Y)	21	0.26	CGA(R)	36	2.62
GUC(V)	5	0.14	UAA	0	0	CGG(R)	1	0.07
GUA(V)	61	1.67	UAG	0	0	AUG(S1)	13	0.29
GUG(V)	3	0.08	CAU(H)	63	1.8	AGC(S1)	1	0.02
UCU(S2)	125	2.82	CAC(H)	7	0.2	AGA(S1)	99	2.24
UCC(S2)	15	0.34	CAA(Q)	61	1.94	AGG(S1)	1	0.02
UCA(S2)	99	2.24	CAG(Q)	2	0.06	GGU(G)	44	0.93
UCG(S2)	1	0.02	AAU(N)	214	1.86	GGC(G)	1	0.02
CCU(P)	76	2.45	AAC(N)	16	0.14	GGA(G)	127	2.67
						GGG(G)	18	0.38

Table S3. The length of 13 PCGs in Tetratomidae species.

	nd2	cox1	cox2	atp8	atp6	cox3	nad3	nad5	nad4	nad4L	nad6	cytb	nad1
P. kochi	1008	1534	688	156	672	789	354	1713	1333	288	507	1137	951
T. fungorum	996	1543	682	156	672	786	354	1645	1333	264	498	1128	948

Table S4. The nonsynonymous (Ka) and synonymous (Ks) of the mitogenome of *Penthe kochi*.

	nd2	cox1	cox2	atp8	atp6	cox3	nad3	nad5	nad4	nad4L	nad6	cytb	nad1
Ka	0.2731	0.7	0.0838	0.2714	0.1148	0.1294	0.1725	0.2585	0.2039	0.2130	0.3334	0.1213	0.1294
Ks	0.8885	0.9200	0.6765	0.5021	0.8625	0.8024	0.7654	0.0742	0.5341	0.4598	0.4643	0.7477	0.4304
Ka/Ks	0.3074	0.0761	0.1234	0.5405	0.1331	0.1613	0.2254	3.4839	0.3818	0.4634	0.7181	0.1622	0.3007

Table S5. The A+T content of Tetratomidae mitogenome.

	Full genome	PCGs	rRNAs	tRNAs	CR
P. kochi	80.60%	78.99%	82.52%	80.08%	87.54%
T. fungorum	77.77%	76.13%	82.88%	80.11%	87.55%

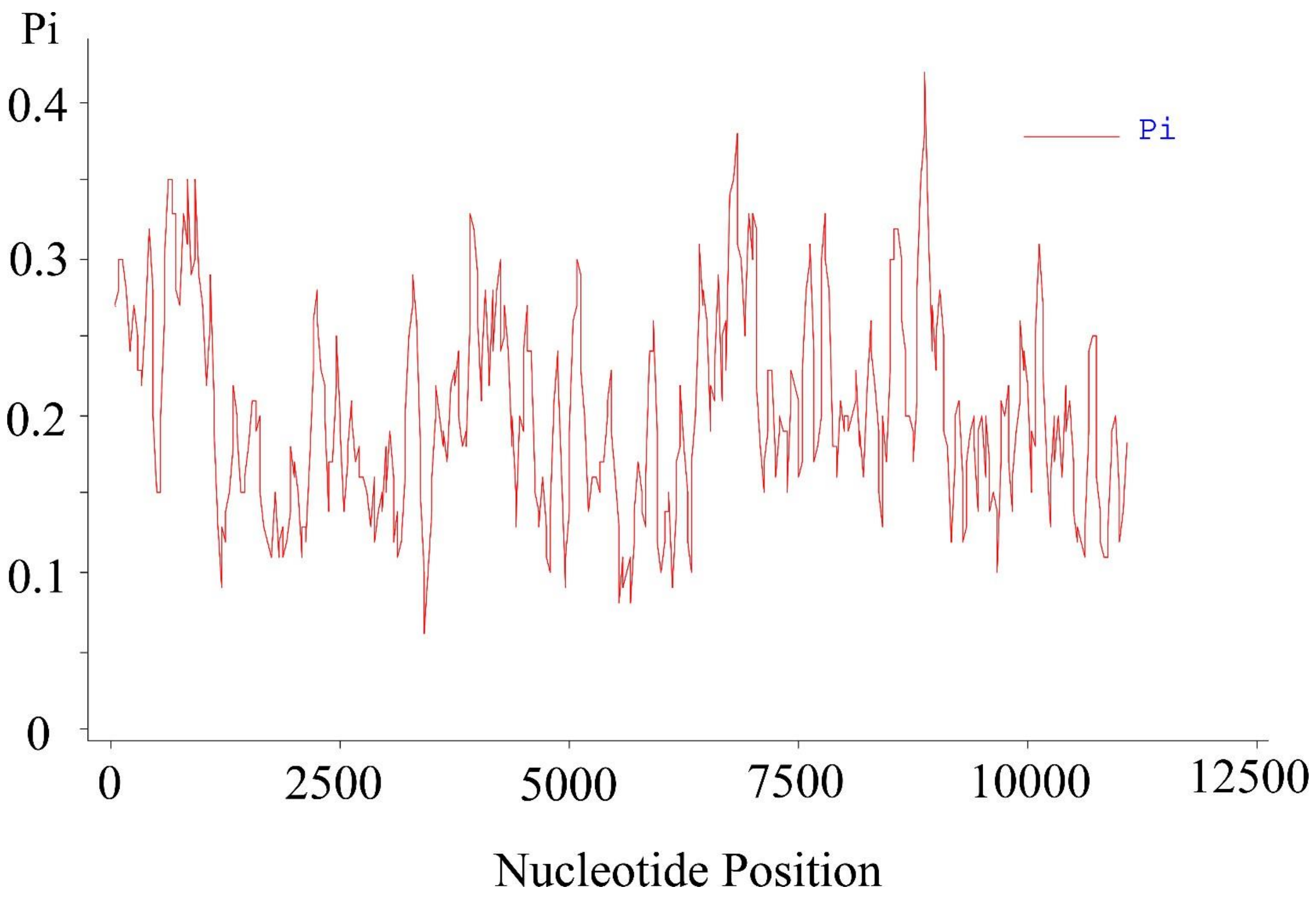

Figure S1. The nucleotide diversity of 13 PCGs of *Penthe kochi*.
